# Effect of simvastatin in the autonomic system is dependent on the increased gain/sensitivity of the baroreceptors

**DOI:** 10.1002/phy2.45

**Published:** 2013-08-22

**Authors:** Edson D Moreira, Cristiano T Mostarda, Ivana C Moraes-Silva, Janaina B Ferreira, Fernando dos Santos, Silvia Lacchini, Kátia De Angelis, Bruno Rodrigues, Maria Cláudia Irigoyen

**Affiliations:** 1 Experimental Hypertension Laboratory, Heart Institute (InCor), University of Sao Paulo Medical SchoolSao Paulo, Brazil; 2 Hospital Universitário Materno Infantil, Federal University of Maranhão (UFMA)Sao Luiz-Maranhão, Brazil; 3 Department of Anatomy, Institute of Biomedical Sciences, University of Sao PauloSao Paulo, Brazil; 4 Translational Physiology Laboratory, Nove de Julho University (UNINOVE)Sao Paulo, Brazil; 5 Human Movement Laboratory, Sao Judas Tadeu University (USJT)Sao Paulo, Brazil

**Keywords:** Baroreceptor function, baroreflex sensitivity, Hypertension, statins, sympathetic modulation

## Abstract

A number of mechanisms have been proposed to explain the pleiotropic effect of statin therapy to reduce sympathetic outflow in cardiovascular disease. We tested the hypothesis that statin treatment could improve baroreflex gain-sensitivity triggered by morphological adaptations in the mechanoreceptor site, thus reducing sympathetic activity, regardless of arterial pressure (AP) level reduction. Male spontaneously hypertensive rats (SHR) were divided into control (SHR, *n* = 8) and SHR-simvastatin (5 mg/kg/day, for 7 days) (SHR-S, *n* = 8). After treatment, AP, baroreflex sensitivity (BRS) in response to AP-induced changes, aortic depressor nerve activity, and spectral analyses of pulse interval (PI) and AP variabilities were performed. Internal and external carotids were prepared for morphoquantitative evaluation. Although AP was similar between groups, sympathetic modulation, represented by the low frequency band of PI (SHR: 6.84 ± 3.19 vs. SHR-S: 2.41 ± 0.96 msec^2^) and from systolic AP variability (SHR: 3.95 ± 0.36 vs. SHR-S: 2.86 ± 0.18 mmHg^2^), were reduced in treated animals. In parallel, simvastatin induced an increase of 26% and 21% in the number of elastic lamellae as well as a decrease of 9% and 25% in the carotid thickness in both, external and internal carotid, respectively. Moreover, improved baroreceptor function (SHR: 0.78 ± 0.03 vs. SHR-S: 1.06 ± 0.04% mv/mmHg) was observed in addition to a 115% increase in aortic depressor nerve activity in SHR-S rats. Therefore, our data suggest that the reduction of sympathetic outflow in hypertension by simvastatin treatment may be triggered by structural changes in the carotid arteries and increased BRS in response to an improvement of the baroreceptors discharge and consequently of the afferent pathway of the baroreflex arch.

## Introduction

Hypertension, a major risk factor in the development of cardiovascular disease, is often associated with alterations in blood pressure control mechanisms. It is well known that chronic elevation of blood pressure alters baroreceptor sensitivity, and induces end organ damage (Andresen and Yang 1989; Grassi 2010). In addition, several studies have suggested that a depressed baroreflex activity is a strong predictor of cardiac mortality (La Rovere and Bigger 1998; La Rovere et al. 2013).

Classically, HMG-CoA reductase inhibitors, or statins, are used to manage dyslipidemia, but they also present beneficial effects beyond lipid lowering; for instance, blood pressure reduction (Sposito et al. 1999; Wassmann et al. 2001; Borghi et al. 2002) in central and peripheral sympathetic activity (Gomes et al. 2010; Deo et al. 2012), and improvement in antiinflammatory responses (Bu et al. 2011). Furthermore, in hypertensive Dahl salt-sensitive rats, treatment with atorvastatin prevented vascular endothelial nitric oxide synthase (eNOS) downregulation and inhibited oxidative stress in the aorta, contributing to the protection against end organ injury in salt-sensitive hypertension (Zhou et al. 2004).

One of the most remarkable pleiotropic effects of statins may be the increase in nitric oxide synthase (NOS) bioactivity, which in turn improves endothelial function (O'Driscoll et al. 1997). Indeed, Rossoni et al. (2011) have demonstrated that simvastatin acutely increases NO phosphorylation in isolated resistance arteries, even in control rats. This statin-related increase in NO bioavailability statins also positively impacts the autonomic nervous system function and baroreflex control of blood pressure by a central nervous system-induced mechanism (Gao et al. 2005; Kishi and Hirooka 2010). Previous attempts to study the mechanisms behind simvastatin-induced cardiac autonomic control improvement in heart failure have shown that not only NO but also AT1 receptors and nicotinamide adenine dinucleotide phosphate oxidase are modulated and could account for the reduction in peripheral sympathetic activity (Gao et al. 2005).

Nevertheless, other peripheral candidate mechanisms may be behind the improved control of blood pressure observed in statin-treated patients. Therefore, we carried out the present study to investigate whether simvastatin treatment, which does not alter resting blood pressure levels, is able to improve autonomic control of the heart and vessels, as well as the baroreflex sensitivity (BRS) and function in hypertensive rats. We tested whether short-term statin treatment could lead to an improvement in baroreflex gain triggered by morphological adaptations in the mechanoreceptor site and to a reduction in sympathetic activity, regardless of any reduction in arterial pressure (AP) levels.

## Methods

### Animals

Sixteen male spontaneously hypertensive rats (SHR) (Federal University of Sao Paulo, Brazil), weighing from 200 to 250 g, were fed standard laboratory chow and water ad libitum in a temperature-controlled room (22°C) with a dark–light cycle of 12/12 h. All procedures were performed in accordance with the *Guidelines for the Care and Use of Laboratory Animals*, published by the National Institutes of Health (NIH Publication No. 85-23, Revised 1996).

The rats were randomly distributed in two groups: SHR (*n* = 8) and SHR treated with simvastatin (SHR-S) (*n* = 8). Simvastatin (lot no SIM0400916-1 Galena, Campinas, SP, Brazil) was dissolved in distilled water and given by gavage during 7 days at a dose of 5 mg/kg/day. A previous study of our laboratory showed that this treatment regimen do not significantly alter AP (data not showed).

### Cardiovascular assessments

After the 7 days of treatment, two catheters filled with 0.06 mL saline were implanted in anesthetized rats (80 mg/kg ketamine and 12 mg/kg xylazine) into the femoral artery and vein (PE-10) for direct measurements of AP and drug administration, respectively. Rats were studied 48 h after catheter placement; the rats were conscious and allowed to move freely in the cage during the experiments. The arterial cannula was connected to a strain-gauge transducer (Blood Pressure XDCR, Kent© Scientific, Litchfield, CT), and AP signals were recorded over a 20-min period by a microcomputer equipped with an analog-to-digital converter board (using a Dataq Instruments DI-720, 16-bit measurement resolution, 2 kHz sampling rate, Springfield, OH). The software used for the acquisition was WINDAQ/PRO (Dataq Instruments, Springfield, OH) waveform recording software. The recorded data were analyzed on a beat-to-beat basis to quantify changes in mean AP (MAP) and heart rate (HR).

In order to evaluate BRS, increasing doses of phenylephrine (0.25–1 μg/animal) and sodium nitroprusside (0.5–2 μg/animal) were given as sequential bolus injections (0.1 mL) to produce AP responses ranging from 5 to 40 mmHg. A 3- to 5-min interval between doses was necessary for AP to return to its baseline. Peak increases or decreases in MAP after phenylephrine or sodium nitroprusside injection and the corresponding peak reflex changes in HR were recorded for each dose of the drug. BRS was evaluated by a mean index through a relationship between changes in HR and in MAP, allowing separate analysis of reflex bradycardia and tachycardia. The mean index was expressed as bpm/mmHg (Farah et al. 1999).

### Power spectral analysis

Spectral analysis of systolic AP (SAP) and pulse interval (PI) were analyzed by autoregressive parametric spectral method that automatically provide the number, center frequency, and power of each oscillatory component. In brief, a derivative-threshold algorithm provides the continuous series PI (tachogram). From the SAP signal, the systogram was created through the beat-to-beat systolic pressure. The low frequencies (0.20–0.75 Hz) and high frequencies (0.75–3.00 Hz) spectral components of PI and SAP were expressed in absolute (ms², mmHg²) and in normalized units (nu). The normalized units were obtained by calculating the power of low frequency (LF) and high frequency (HF) correlating each to the total power, after subtracting the power of the very low-frequency component (frequencies of <0.2 Hz) (Soares et al. 2004).

### Baroreceptor gain-sensitivity

The recording of the whole-nerve activity of the aortic depressor nerve for aortic baroreceptor gain-sensitivity evaluation was performed in anesthetized rats (sodium pentobarbital, 30 mg/kg, i.p.) after 7 days of Simvastatin treatment. Platinum bipolar electrodes (0.050 mm thickness) with high-input impedance and small interelectrode distance (1.2 mm between electrodes) were fixed at the aortic depressor nerve, protected with a lightweight silicon impression material (Wacker Sil-Gel 604, Wacker-Chemie, Munich, Germany), and continuously displayed on an oscilloscope (5115 Tektronix Storage Oscilloscope, Heerenveen, Netherlands).

The nerve action potentials were amplified (5–10 kHz of gain; AN502 Differential Amplifier, Tektronix, OR), filtered (bandwidth 0.1–3.0 kHz), and the full-wave activity were rectified, and further integrated by using the pulse pressure as a trigger on a beat-to-beat basis. Both aortic depressor nerve activity and AP were sampled at 10 kHz (DataQ Instruments, Inc., OH).

The aortic baroreceptor gain-sensitivity was evaluated by two different protocols: during spontaneous and induced changes of blood pressure (phenylephrine, 5 μg/kg and sodium nitroprusside, 5 μg/kg) by drug infusion via the femoral vein. To permit comparison of the baroreceptor discharges in different rats, nerve activity was normalized as a percentage of the maximal discharge at higher AP levels in the control animals (expressed as % mmHg^−1^), as previously described by our group (Brum et al. 2000; Soares et al. 2004; Rondon et al. 2006).

Two approaches were used to analyze the AP-nerve activity relationship. The baroreceptor gain-sensitivity was evaluated by linear regression and by the full range of AP variations. The full SAP range for baroreceptor activation, defined by the difference between SP_th_ (systolic pressure at which the baroreceptors initiated firing) and SP_st_ (the pressure level at which continuous baroreceptor discharge was achieved during a rapid increase in AP) was used to calculate the index of sensitivity expressed as%.mv/mm/Hg. Moreover, baroreceptor gain-sensitivity was evaluated by fitting a linear regression describing the relationship of the mean changes in the nerve discharge obtained during SAP variations ranges of 2 mmHg. The average slope of the liner regression was considered as an index of baroreceptor gain-sensitivity (Brum et al. 2000; Rondon et al. 2006).

### Carotid morphometric evaluations

To evaluate carotid histology, hearts were arrested in diastole by perfusion with a NaCl (0.9%) plus 14 mmol/L KCl solution, followed by buffered formalin (4%) for tissue fixation. The neck was harvested to expose the neurovascular bundle of the neck. The entire bundle was dissected with trachea and associated muscles, processed, and embedded in paraplast. Sections of 5 μm were obtained in the region of carotid bifurcation, and stained with Weigert for elastic lamellae and thickness evaluations. Histo-morphometric analyses were performed blinded regarding the identity of experimental groups (Leica Imaging Systems, Wetzlar, Germany) and analysis were used to measure carotid media area and elastic lamellae counting (Image Quant-Leica, Leica Microsystems, Wetzlar, Germany). Internal and external areas were measured and elastic lamellae were counted from 5 slices per animal.

### Statistical analysis

Data are reported as means ± SEM. Student's *t*-test was used to compare the groups. Pearson correlation was used to determine association among variables. The significance level was established as *P* < 0.05.

## Results

### Hemodynamics, BRS, and time domain cardiovascular autonomic modulation

Simvastatin did not significantly alter SAP, diastolic AP (DAP), or MAP in this model (Table 1). Besides, there were no differences in time domain AP variability, as expressed by SAP variance (VAR SAP) between groups. However, the SHR-S group showed increased HR variability, [as] expressed by the PI variance (VAR PI). In addition, BRS estimated by the response to vasoactive drugs was higher in the treated group (SHR-S) when compared to the control (SHR). Indeed, the improvement in BRS was demonstrated by the increase of both reflex tachycardia (ITR) and reflex bradycardia (IBR) indexes (Table 1).

**Table 1:** Hemodynamic and autonomic evaluations in awake spontaneously hypertensive rats (SHR) and SHR-S groups

	SHR	SHR-S
SAP (mmHg)	206 ± 3.98	198 ± 1.50
DAP (mmHg)	138 ± 0.60	136 ± 2.91
MAP (mmHg)	170 ± 3.79	165 ± 0.64
HR (bpm)	338 ± 9.65	341 ± 9.02
PI (ms)	176 ± 7.54	178 ± 6.58
VAR SAP (mmHg²)	37.42 ± 4.10	30.20 ± 3.12
VAR PI (ms²)	23.34 ± 3.46	41.62 ± 6.10*
ITR (bpm/mmHg)	0.95 ± 0.11	1.39 ± 0.12*
IBR (bpm/mmHg)	0.67 ± 0.02	1.11 ± 0.13*

Values are mean ± SEM. Results of the systolic arterial pressure (SAP), systolic arterial pressure variance (VARSAP), mean arterial pressure (MAP), diastolic arterial pressure (DAP), heart rate (HR), pulse interval (PI), pulse interval variance (VAR PI), index of reflex tachycardia (ITR), and index of reflex bradycardia (IBR).Student *t*-test, *P < 0.05 versus SHR group.

### SAP and HR variability in frequency domain

Table 2 shows the spectral analysis of HR and SAP variability. Simvastatin significantly reduced sympathetic modulation of the vessels, represented by the LF component of SAP. Concerning HR variability, the treatment reduced sympathetic modulation of the heart, as demonstrated by the reduction of both absolute and normalized power of the LF component of PI. In addition, simvastatin increased the normalized HF component of HR variability in treated animals when compared with untreated SHR, demonstrating that simvastatin treatment improved vagal modulation of cardiac autonomic modulation, thus reducing the sympathovagal balance (LF/HF) in the SHR-S group when compared with SHR group.

**Table 2:** Frequency domain analysis of the heart rate and blood pressure variability in spontaneously hypertensive rats (SHR) and SHR-S groups

	SHR	SHR-S
PI (tachogram)		
LF (ms²)	6.84 ± 3.19	2.41 ± 0.96*
LF (NU)	30 ± 3.65	18 ± 1.72*
HF (ms²)	18.39 ± 4.53	16.32 ± 3.07
HF (NU)	70 ± 3.65	82 ± 1.72*
LF/HF	0.45 ± 0.10	0.24 ± 0.02*
SAP(systogram)		
LF(z²)	3.95 ± 0.36	2.86 ± 0.18*

Values expressed as mean ± SEM. Results of spectral powers of SAP (systolic arterial pressure) and PI (pulse interval) computed from 0.20 to 3 Hz (total power plot), low-frequency (LF 0.20–0.75 Hz) and high-frequency (HF 0.75–3.00 Hz) bands in absolute (ms^2^) and normalized units (NU), and autonomic balance (LF/HF) in SHR and SHR + S groups. Student's *t*-test, *P < 0.05 versus SHR group.

### Baroreceptor resetting and gain-sensitivity

**Figure 1:**
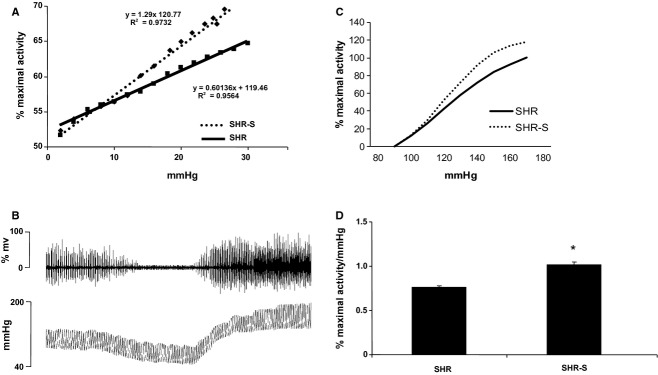
Baroreflex function and sensitivity. (A) Spontaneous aortic depressor nerve activity, expressed by% of maximal activity/mmHg in spontaneously hypertensive rats (SHR) and SHR-S; (B) Representative experiment of baroreceptor function curve showing the changes in aortic activity (top) associated with pulse pressure variation induced by vasoactive drug infusion. (C) Baroreceptor function curve obtained by the aortic activity changes related to induced variations of arterial pressure. (D) Slope of the baroreceptor function curve expressed by%maximal activity/mmHg in SHR and SHR-S, obtained by arterial pressure (AP)-induced changes. Values expressed as mean ± SEM, (A): Pearson Correlation, (B) Student *t*-test, *P < 0.05 versus SHR group.

Simvastatin improved baroreceptor function and sensitivity, regardless of any changes in AP levels and other hemodynamic responses in the treated group. SHR-S presented improved spontaneous aortic nerve activity response to changes of AP levels at rest as expressed by the slope of the line relating activity and AP values (Fig. 1A) and enhanced baroreceptor function, as evaluated by the increase in the percentage of total activity, which represents the electrical potential outflow of the aortic depressor nerve (Fig. 1B).

### Morphometric analyses of carotid

**Figure 2:**
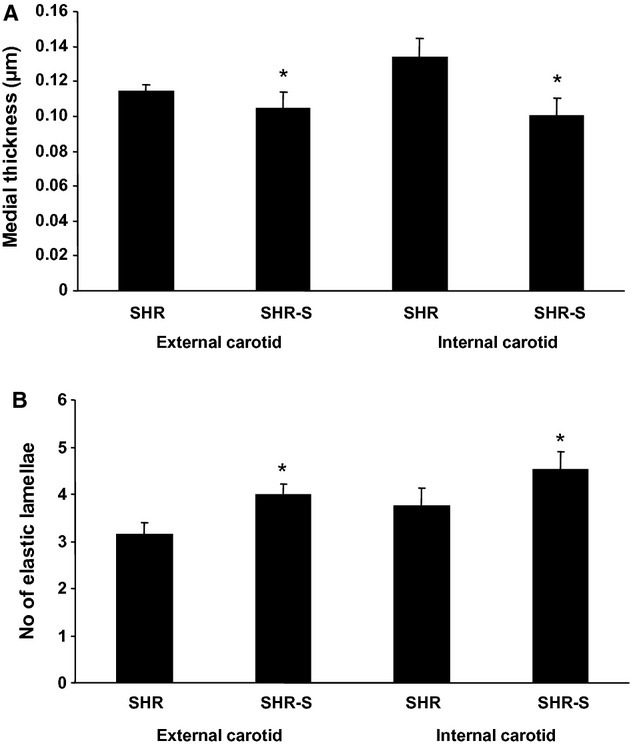
External and Internal carotid morphology. (A) Medial external and internal carotid thickness. (B) Number of elastic lamellae in external and internal carotid. Student *t*-test, *P < 0.05 versus spontaneously hypertensive rats (SHR).

**Figure 3:**
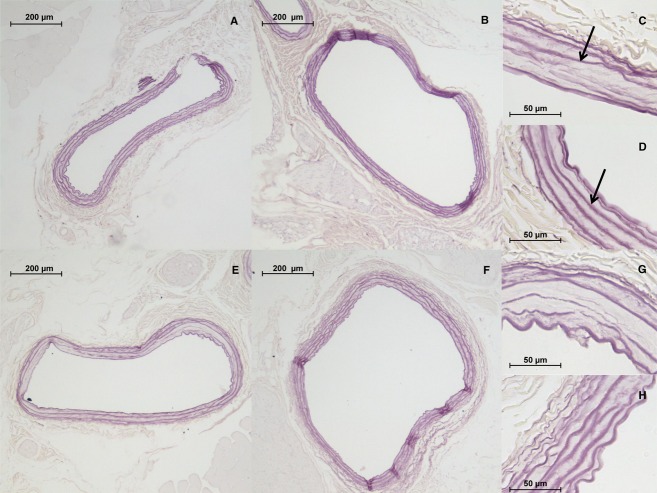
Representative photomicrographies showing morphology of external carotid arteries of spontaneously hypertensive rats (SHR) (A and C) compared with SHR-S (B and D). Note the decrease of arterial thickness and the increase of elastic lamellae number in SHR-S (D), lamellae indicated by arrows). Representative photomicrographies showing morphology of internal carotid arteries of SHR (E and G) compared with SHR-S (F and H). Note also the decrease of arterial thickness and the increase of elastic lamellae number in SHR-S (G).

After treatment with simvastatin, SHR-S presented reduced external (0.115 ± 0.003 vs. 0.104 ± 0.009 μm, *P* = 0.035) and internal (0.134 ± 0.010 vs. 0.101 ± 0.10 μm, *P* < 0.001) carotid thickness (Fig. 2A). In addition, we observed an increase in the number of elastic lamellae in both external (3.16 ± 0.2 vs. 3.99 ± 0.2 lamellae, *P* < 0.001) and internal carotid (3.76 ± 0.36 vs. 4.53 ± 0.36 lamellae, *P* = 0.003) in the SHR-S group when compared with control SHR (Fig. 2B). Representative photomicrographies are presented in Figure 3.

## Discussion

The main findings of the present study were that short-term simvastatin treatment induced an improvement of cardiovascular autonomic control of the heart and vessels in SHR, as demonstrated by a reduction in sympathetic modulation. The mechanisms underlying this reduction seem to be related to the aortic depressor nerve activity enhancement. This enhancement would improve BRS and would lead to better arterial distensibility, as demonstrated by the increase in the number of carotid elastic lamellae and the reduction in intima thickness.

Beyond the potent pharmacologic inhibition of cholesterol biosynthesis of statins, which made this class of drugs so widely prescribed, their potential pleiotropic effects – reduction in AP and sympathetic outflow in cardiovascular disease – have been attracting a growing interest in recent years (Fisher and Fadel 2010).

Pliquett et al. (2003) were the first to provide evidence that statin therapy has a potent modulatory effect on sympathetic tone and autonomic function in an animal model of chronic heart failure. Furthermore, Kishi et al. (2003) have observed a significant reduction in 24 h urinary norepinephrine concentrations in stroke-prone SHR treated with atorvastatin. More recently, in clinical settings, studies with chronic kidney disease patients (Siddiqi et al. 2011) and primary hypertension (Sinski et al. 2009) have demonstrated a reduction in muscle sympathetic nerve activity following statin treatment.

The results of the present study are in line with the experimental and clinical findings. Our study demonstrated that short-term simvastatin treatment reduced low-frequency band of PI and of SAP variability in hypertensive rats, leading to/resulting in a considerable improvement in the sympathovagal balance in treated animals and thus contributing to a better baroreflex function. This finding is in accordance with previous studies which have demonstrated maintenance of blood pressure and HR values accompanied by changes of autonomic components to the heart or to the vessels (Kishi et al. 2003; Sinski et al. 2009; Siddiqi et al. 2011).

Oral treatment with simvastatin has been shown to improve sympathetic outflow and restore arterial baroreflex function in rabbits with chronic heart failure. This is probably accomplished by the inhibition of angiotensin II mechanisms and reactive oxygen species in the rostral ventrolateral medulla (Pliquett et al. 2003; Gao et al. 2005).

Furthermore, Gao et al. (2008) have offered evidence that direct intracerebroventricular infusion of simvastatin has a beneficial effect on sympathetic nerve activity and baroreflex function in chronic heart failure animals. This improvement was accompanied by an upregulation of neuronal NOS protein expression in the rostral ventrolateral medulla.

Similarly, Kishi et al. (2003) have demonstrated that a 30-day atorvastatin treatment in hypertensive rats led to an upregulation of brainstem eNOS and significant reductions in urinary noradrenaline excretion and AP. Furthermore, the oral administration of atorvastatin inhibited the activation of the sympathetic nervous system and improved the baroreflex control in hypertensive rats via reduction in reactive oxygen species in the rostral ventrolateral medulla of hypertensive rats (Kishi et al. 2008, 2009). In mice with sympathetic hyperactivity induced by myocardial infarction, the increase in eNOS expression in the nucleus tractus solitarius significantly attenuated the enhanced sympathetic drive (Sakai et al. 2005).

In order to expand our knowledge about the mechanisms underlying the reduction in sympathetic nervous activity in hypertension condition, we also hypothesized that an alteration in loco might influence the sensitivity and function of baroreceptors, the most important short-term control mechanism of AP regulation altered by hypertension. Indeed, the loss of distensibility of the aorta and carotid arteries has been associated with changes in BRS (Hunt et al. 2001; Kaushal and Taylor 2002), as the mechanical stress of the arteries wall would not be able to adequately trigger the mechanoreceptors.

It is worth stressing that most of the studies on the pleiotropic effects of statins, reported AP reduction after treatment. In our study, we chose a simvastatin dose (5 mg/kg/day) which would not induce AP alteration in SHR, in an attempt to isolate the observed responses from the hemodynamic effect, as an AP decrease is by itself a contributor to functional and structural beneficial adaptations. Therefore, we observed that, even with unaltered AP, simvastatin increased the number of elastic lamellae and decreased intima thickness in the internal and external carotid in SHR.

In this sense, the mechanism associated with a reduction in sympathetic modulation of the heart and vessels by simvastatin treatment may also be associated with positive morphological changes in carotid and may be further extended to other vessels, as the aorta, improving artery mechanical distension, and triggering more efficiently the baroreflex signal to the nucleus of the solitary tract in the bulb. In this way, the central sympathetic response to the heart and peripheral vasculature would be reduced. However, a question that needs to be answered concerns the underlying mechanisms of improved vascular remodeling by simvastatin treatment.

It has been demonstrated that atorvastatin reduced the collagen content and improved the alterations of elastin structure induced by angiotensin II in resistance arteries of rats (Briones et al. 2009). Indeed, statins can directly induce eNOS by activating phosphatidylinositol-3-kinase(PI3K)/protein kinase B/Akt signals (Kureishi et al. 2000) that inhibit the Rho A/Rho kinase signaling and upregulate eNOS expression (Rikitake and Liao 2005; Balakumar et al. 2012). In addition, statins abrogate caveolin-1 expression, which is a negative eNOS regulator (Plenz et al. 2004; Balakumar et al. 2012).

In large arteries, statin treatment decreased perivascular coronary artery fibrosis (Kishi et al. 2008), oxidative stress in the aorta (Zhou et al. 2004), and extracellular matrix deposition in the heart (Zhai et al. 2008) and rat aorta (Rupérez et al. 2007). In fact, a recent meta-analysis involving 6317 individuals has demonstrated that statin therapy was efficient in reducing common carotid intima-media thickness (Balakumar et al. 2012). Furthermore, Ratchford et al. (Plenz et al. 2004) have observed decreased stiffness and increased distensibility in carotids of humans after a 30-day atorvastatin treatment, regardless of cholesterol lowering.

On the other hand, we cannot rule out the hypothesis that a central action of simvastatin reduced efferent sympathetic discharge (as evaluated by LF component of the heart and vessels), modulating oxidative stress, inflammation, and, consequently structural changes in carotid artery. In fact, the increase in AP variability, without any changes in AP levels, triggered negative morphological changes in left and right ventricles (Moraes-Silva et al. 2010; Flues et al. 2012), as well as in the vessels (Ceroni et al. 2009) of sinoaortic denervated animals. Additionally, previous studies have demonstrated that autonomic nervous system can modulate inflammation and oxidative stress (Pavlov and Tracey 2012). Recently, in a study involving simvastatin treatment associated or not with exercise training, we have observed that the cardiac lipoperoxidation was negatively related to the vagal effect, and positively correlated to the sympathetic effect (Bernardes et al. 2013).

In this sense, the reduction of sympathetic component (LF band) to the heart and vessels may reduce inflammation and oxidative stress, reducing carotid histopathological changes, and as such improving baroreflex afference. In other words, regardless of the mechanisms involved in arterial structural changes, the increase in baroreceptor afferent discharge in treated animals may account for the reduced peripheral sympathetic modulation.

In summary, the data of the present investigation suggest that short-term simvastatin treatment induce improvement in PI variability, reduction in sympathetic modulation of the heart and vessels, as well as increase in BRS in SHR, regardless of changes in the AP. The possible mechanism associated with this autonomic improvement was a structural change in the carotid arteries, leading to an adequate trigger of the mechanoreceptors and an improved afferent pathway of the baroreflex arch. Although clinical trials are required to determine whether carotid evaluation may predict cardiovascular autonomic control, and whether these pleiotropic effects of statins may prevent cardiovascular outcomes, this study lends support to the possible association of arterial morphology to estimate BRS and function, and sympathetic outflow in hypertensive subjects.

## References

[b1] Andresen MC, Yang M (1989). Arterial baroreceptor resetting: contributions of chronic and acute processes. Clin. Exp. Pharmacol. Physiol. Suppl.

[b2] Balakumar P, Kathuria S, Taneja G, Kalra S, Mahadevan N (2012). Is targeting eNOS a key mechanistic insight of cardiovascular defensive potentials of statins?. J. Mol. Cell. Cardiol.

[b3] Bernardes N, Brito JO, Fernandes TG, Llesuy SF, Irigoyen MC, Bêllo-Klein A (2013). Pleiotropic effects of simvastatin in physically trained ovariectomized rats. Braz. J. Med. Biol. Res.

[b4] Borghi C, Dormi A, Veronesi M, Immordino V, Ambrosioni E (2002). Use of lipid-lowering drugs and blood pressure control in patients with arterial hypertension. J. Clin. Hypertens (Greenwich).

[b5] Briones AM, Rodríguez-Criado N, Hernanz R, García-Redondo AB, Rodrigues-Díez RR, Alonso MJ (2009). Atorvastatin prevents angiotensin II-induced vascular remodeling and oxidative stress. Hypertension.

[b6] Brum PC, Moreira GJ, Da Silva ED, Ida F, Negrao CE, Krieger EM (2000). Exercise training increases baroreceptor gain sensitivity in normal and hypertensive rats. Hypertension.

[b7] Bu DX, Griffin G, Lichtman AH (2011). Mechanisms for the anti-inflammatory effects of statins. Curr. Opin. Lipidol.

[b8] Ceroni A, Chaar LJ, Bombein RL, Michelini LC (2009). Chronic absence of baroreceptor inputs prevents training-induced cardiovascular adjustments in normotensive and spontaneously hypertensive rats. Exp. Physiol.

[b9] Deo SH, Fisher JP, Vianna LC, Kim A, Chockalingam A, Zimmerman MC (2012). Statin therapy lowers muscle sympathetic nerve activity and oxidative stress in patients with heart failure. Am. J. Physiol. Heart Circ. Physiol.

[b10] Farah VM, Moreira ED, Pires MD, Irigoyen MC, Krieger EM (1999). Comparison of three methods for the determination of baroreflex sensitivity in conscious rats. Braz. J. Med. Biol. Res.

[b11] Fisher JP, Fadel PJ (2010). Therapeutic strategies for targeting excessive central sympathetic activation in human hypertension. Exp. Physiol.

[b12] Flues K, Moraes-Silva IC, Mostarda C, Souza PR, Diniz GP, Moreira ED (2012). Cardiac and pulmonary arterial remodeling after sinoaortic denervation in normotensive rats. Auton. Neurosci.

[b13] Gao L, Wang W, Li YL, Schultz HD, Liu D, Cornish KG (2005). Simvastatin therapy normalizes sympathetic neural control in experimental heart failure: roles of angiotensin II type 1 receptors and NAD(P)H oxidase. Circulation.

[b14] Gao L, Wang W, Zucker IH (2008). Simvastatin inhibits central sympathetic outflow in heart failure by a nitric-oxide synthase mechanism. J. Pharmacol. Exp. Ther.

[b15] Gomes ME, Tack CJ, Verheugt FW, Smits P, Lenders JW (2010). Sympathoinhibition by atorvastatin in hypertensive patients. Circ. J.

[b16] Grassi G (2010). Sympathetic neural activity in hypertension and related diseases. Am. J. Hypertens.

[b17] Hunt BE, Farquhar WB, Taylor JA (2001). Does reduced vascular stiffening fully explain preserved cardiovagal baroreflex function in older, physically active men?. Circulation.

[b18] Kaushal P, Taylor JA (2002). Inter-relations among declines in arterial distensibility, baroreflex function and respiratory sinus arrhythmia. J. Am. Coll. Cardiol.

[b19] Kishi T, Hirooka Y (2010). Sympathoinhibitory effects of atorvastatin in hypertension. Circ. J.

[b20] Kishi T, Hirooka Y, Mukai Y, Shimokawa H, Takeshita A (2003). Atorvastatin causes depressor and sympatho-inhibitory effects with upregulation of nitric oxide synthases in stroke-prone spontaneously hypertensive rats. J. Hypertens.

[b21] Kishi T, Hirooka Y, Shimokawa H, Takeshita A, Sunagawa K (2008). Atorvastatin reduces oxidative stress in the rostral ventrolateral medulla of stroke-prone spontaneously hypertensive rats. Clin. Exp. Hypertens.

[b22] Kishi T, Hirooka Y, Konno S, Sunagawa K (2009). Atorvastatin improves the impaired baroreflex sensitivity via anti-oxidant effect in the rostral ventrolateral medulla of SHRSP. Clin. Exp. Hypertens.

[b23] Kureishi Y, Luo Z, Shiojima I, Bialik A, Fulton D, Lefer DJ (2000). The HMG-CoA reductase inhibitor simvastatin activates the protein kinase Akt and promotes angiogenesis in normocholesterolemic animals. Nat. Med.

[b24] La Rovere MT, Bigger JT (1998). Marcus FI, Mortara A, Schwartz PJ. Baroreflex sensitivity and heart-rate variability in prediction of total cardiac mortality after myocardial infarction. ATRAMI (Autonomic Tone and Reflexes After Myocardial Infarction) Investigators. Lancet.

[b25] La Rovere MT, Pinna GD, Maestri R, Sleight P (2013). Clinical value of baroreflex sensitivity. Neth. Heart J.

[b26] Moraes-Silva IC, Mostarda RN, De La Fuente C, Rosa K, Flues K, Damaceno-Rodrigues NR (2010). Baroreflex deficit blunts exercise training-induced cardiovascular and autonomic adaptations in hypertensive rats. Clin. Exp. Pharmacol. Physiol. Suppl.

[b27] O'Driscoll G, Green D, Taylor RR (1997). Simvastatin, an HMG-coenzyme A reductase inhibitor, improves endothelial function within 1 month. Circulation.

[b28] Pavlov VA, Tracey KJ (2012). The vagus nerve and the inflammatory reflex–linking immunity and metabolism. Nat. Rev. Endocrinol.

[b29] Plenz GA, Hofnagel O, Robenek H (2004). Differential modulation of caveolin-1 expression in cells of the vasculature by statins. Circulation.

[b30] Pliquett RU, Cornish KG, Zucker IH (2003). Statin therapy restores sympathovagal balance in experimental heart failure. J. Appl. Physiol.

[b31] Rikitake Y, Liao JK (2005). Rho GTPases, statins, and nitric oxide. Circ. Res.

[b32] Rondon E, Brasileiro-Santos MS, Moreira ED, Rondon MU, Mattos KC, Coelho MA (2006). Exercise training improves aortic depressor nerve sensitivity in rats with ischemia-induced heart failure. Am. J. Physiol. Heart Circ. Physiol.

[b33] Rossoni LV, Wareing M, Wenceslau CF, Al-Abri M, Cobb C, Austin C (2011). Acute simvastatin increases endothelial nitric oxide synthase phosphorylation via AMP-activated protein kinase and reduces contractility of isolated rat mesenteric resistance arteries. Clin. Sci. (Lond).

[b34] Rupérez M, Rodrigues-Díez R, Blanco-Colio LM, Sánchez-López E, Rodríguez-Vita J, Esteban V (2007). HMG-CoA reductase inhibitors decrease angiotensin II-induced vascular fibrosis: role of RhoA/ROCK and MAPK pathways. Hypertension.

[b35] Sakai K, Hirooka Y, Shigematsu H, Kishi T, Shimokawa H, Takeshita A (2005). Overexpression of eNOS in brain stem reduces enhanced sympathetic drive in mice with myocardial infarction. Am. J. Physiol. Heart Circ. Physiol.

[b36] Siddiqi L, Joles JA, Oey PL, Blankestijn PJ (2011). Atorvastatin reduces sympathetic activity in patients with chronic kidney disease. J. Hypertens.

[b37] Sinski M, Lewandowski J, Ciarka A, Bidiuk J, Abramczyk P, Dobosiewicz A (2009). Atorvastatin reduces sympathetic activity and increases baroreceptor reflex sensitivity in patients with hypercholesterolaemia and systemic arterial hypertension. Kardiol. Pol.

[b38] Soares PP, Ushizima AC, da Nobrega MR, Irigoyen MC (2004). Cholinergic stimulation with pyridostigmine increases heart rate variability and baroreflex sensitivity in rats. Auton. Neurosci.

[b39] Sposito AC, Mansur AP, Coelho OR, Nicolau JC, Ramires JA (1999). Additional reduction in blood pressure after cholesterol-lowering treatment by statins (lovastatin or pravastatin) in hypercholesterolemic patients using angiotensin-converting enzyme inhibitors (enalapril or lisinopril). Am. J. Cardiol.

[b40] Wassmann S, Laufs U, Baumer AT, Muller K, Ahlbory K, Linz W (2001). HMG-CoA reductase inhibitors improve endothelial dysfunction in normocholesterolemic hypertension via reduced production of reactive oxygen species. Hypertension.

[b41] Zhai Y, Gao X, Wu Q, Peng L, Lin J, Zuo Z (2008). Fluvastatin decreases cardiac fibrosis possibly through regulation of TGF-beta(1)/Smad 7 expression in the spontaneously hypertensive rats. Eur. J. Pharmacol.

[b42] Zhou MS, Jaimes EA, Raij L (2004). Atorvastatin prevents end-organ injury in salt-sensitive hypertension: role of eNOS and oxidant stress. Hypertension.

